# Digital Health Professions Education in the Field of Pediatrics: Systematic Review and Meta-Analysis by the Digital Health Education Collaboration

**DOI:** 10.2196/14231

**Published:** 2019-09-25

**Authors:** Serena Brusamento, Bhone Myint Kyaw, Penny Whiting, Li Li, Lorainne Tudor Car

**Affiliations:** 1 Department of Primary Care and Public Health School of Public Health Imperial College London London United Kingdom; 2 Centre for Population Health Sciences Lee Kong Chian School of Medicine Nanyang Technological University Singapore Singapore; 3 Population Health Sciences, Bristol Medical School University of Bristol Bristol United Kingdom; 4 Office of Medical Education Lee Kong Chian School of Medicine Nanyang Technological University Singapore Singapore; 5 Family Medicine and Primary Care Lee Kong Chian School of Medicine Nanyang Technological University Singapore Singapore

**Keywords:** digital education, randomized controlled trials, pediatrics, systematic review, meta-analysis, traditional learning, high-fidelity mannequins

## Abstract

**Background:**

Reducing childhood morbidity and mortality is challenging, particularly in countries with a shortage of qualified health care workers. Lack of trainers makes it difficult to provide the necessary continuing education in pediatrics for postregistration health professionals. Digital education, teaching and learning by means of digital technologies, has the potential to deliver medical education to a large audience while limiting the number of trainers needed.

**Objective:**

The goal of the research was to evaluate whether digital education can replace traditional learning to improve postregistration health professionals’ knowledge, skills, attitudes, and satisfaction and foster behavior change in the field of pediatrics.

**Methods:**

We completed a systematic review of the literature by following the Cochrane methodology. We searched 7 major electronic databases for articles published from January 1990 to August 2017. No language restrictions were applied. We independently selected studies, extracted data, and assessed risk of bias, and pairs of authors compared information. We contacted authors of studies for additional information if necessary. All pooled analyses were based on random effects models. We included individually or cluster randomized controlled trials that compared digital education with traditional learning, no intervention, or other forms of digital education. We assessed the quality of evidence using the Grading of Recommendations, Assessment, Development, and Evaluations (GRADE) criteria.

**Results:**

Twenty studies (1382 participants) were included. Participants included pediatricians, physicians, nurses, and midwives. Digital education technologies were assessed including high-fidelity mannequins (6 studies), computer-based education (12 studies), mobile learning (1 study), and virtual reality (1 study). Most studies reported that digital education was either as effective as or more effective than the control intervention for outcomes including skill, knowledge, attitude, and satisfaction. High-fidelity mannequins were associated with higher postintervention skill scores compared with low-fidelity mannequins (standardized mean difference 0.62; 95% CI 0.17-1.06; moderate effect size, low-quality evidence). One study reported physician change in practicing behavior and found similar effects between offline plus online digital education and no intervention. The only study that assessed impact on patient outcome found no difference between intervention and control groups. None of the included studies reported adverse or untoward effects or economic outcomes of the digital education interventions. The risk of bias was mainly unclear or high. The quality of evidence was low due to study inconsistencies, limitations, or imprecision across the studies.

**Conclusions:**

Digital education for postregistration health professions education in pediatrics is at least as effective as traditional learning and more effective than no learning. High-fidelity mannequins were found to be more effective at improving skills than traditional learning with low-fidelity mannequins. Computer-based offline/online digital education was better than no intervention for knowledge and skill outcomes and as good as traditional face-to-face learning. This review highlights evidence gaps calling for more methodologically rigorous randomized controlled trials on the topic.

**Trial Registration:**

PROSPERO CRD42017057793; https://tinyurl.com/y5q9q5o6

## Introduction

Reducing childhood morbidity and mortality is a global health priority. Mortality remains high in many low- and middle-income countries (LMIC), despite improvements achieved as a result of the Millennium Development Goals [1,2]. Reducing childhood mortality and ensuring global access to health care through health workforce development is one of the 17 United Nations Sustainable Development Goals [3-5]. A major factor in reducing childhood morbidity and mortality is the quality of pediatric health care. This is influenced by the skills of the health professionals—physicians, nonphysician clinicians, nurses, and midwives. To provide optimal care, health professionals need continuous, high-quality, and up-to-date education [6]. Lack of access to learning resources, coupled with remote locations, limited health professionals, and a need for the ongoing provision of health services represent significant barriers to health professions education in many settings.

Pediatric health professions education is particularly important due to the unique nature of the diseases, need for timely and appropriate treatment, and the narrow margin of treatment error compared with the adult patients [7]. Additionally, there is a growing demand to educate pediatrics health professionals in certain topics that need timely incorporation/implementation of evidence-based recommendations and guidelines such as delivering updated guidelines on immunizations [8], chemotherapy [9], respiratory infections [10], and neonatal management [11]. Traditional forms of education such as face-to-face or didactic lectures or workshops might not be adequate to address these demands in a timely manner, and digital education can be an alternative option for educating pediatric health professionals as it provides an efficient, timely, and convenient mode for the learners which further helps to improve outcomes [12].

Digital education helps overcome resource, geographical, and time barriers. Computer-based and mobile learning allow learners to access educational materials without time or place restrictions, allowing them to work at their own pace and time from any location [13]. A further advantage of this type of learning is that it generally requires fewer tutors. Unlike face-to-face lectures or tutorials, the number of learners who can participate in this type of education is far greater. Computer-based offline digital education provides increased access to learning materials with limited internet connectivity [14,15]. Mobile learning or mLearning supports learning in a similar way by enhancing the delivery of learning materials without time and place limitation through a handheld mobile device. There are a number of mobile device–based functions such as short message service (SMS or texts), multimedia message service, podcasts, and mobile apps that support the delivery of educational materials based on the needs of learners and learning processes [16,17].

Simulation-based medical education such as training in virtual reality environments and virtual patient scenarios supports creation of 3D virtual world or patient case-based scenarios that are similar to real-life clinical scenarios, designed specifically for health professional training [18-20]. Similarly, training via psychomotor skills trainers such as high- or middle-fidelity mannequins allows for training of different types of psychomotor or technical skills acquisitions such as resuscitation and suturing skills [21-23].

The potential benefits of digital education for health professions education have been evaluated in previous reviews and acknowledged by the World Health Organization [12-14,24-27]. While there are reviews on the use of digital education in specific pediatric fields (eg, emergency or rehabilitation care) and for undergraduate education [28-30], we are unaware of any systematic review assessing the effectiveness of digital education in the field of pediatrics for postregistration health professionals. This review evaluates the effectiveness of different modalities of digital education for postregistration pediatrics health professionals in comparison with traditional learning or other forms of digital education. We assessed the impact of digital education on participants’ knowledge, skills, attitudes, clinical practice, and satisfaction compared with other forms of learning.

## Methods

### Systematic Review

The protocol for the systematic review was registered with PROSPERO [CRD42017057793] [31]. For a detailed description of the methodology, please refer to the methods as described by the Digital Health Education Collaboration [32], a global initiative focused on evaluating the effectiveness of digital health professions education through a series of methodologically robust systematic reviews.

For the purpose of this review, digital education can be defined as “an approach to teaching and learning, representing all or part of the educational model applied, that is based on the use of electronic media and devices as tools for improving access to training, communication and interaction, that facilitates the adaptation of new ways of understanding and developing learning” [33]. Digital education encompasses a variety of learning modalities including computer-based online/offline digital education (online/offline digital education), high-fidelity mannequins, virtual reality environments, virtual patient scenarios, serious gaming and gamification, and mobile learning, etc [15,20,27,34-36]. It is often combined with traditional nondigital learning, known as blended learning. Traditional learning means learning via traditional forms of education such as paper- or textbook–based learning, didactic or face-to-face lectures, tutorials, box trainers, or low-fidelity mannequins.

### Inclusion Criteria

We included studies involving learners who were enrolled in any postregistration health professional or continuing medical education (CME) programs. For this review, postregistration health professional programs can be defined as any type of study after a qualification which is recognized by the relevant governmental or professional bodies that enables the qualification holder entry into or continuation of work in the health care workforce in a more independent or senior role. We also included studies focusing on CME programs that involved the use of digital education to deliver the learning contents. We included all postregistration health professionals listed in the Health Field of Education and Training (091) of the International Standard Classification of Education except professionals from traditional, alternative, and complementary medicine [31].

We included individually or cluster randomized controlled trials (RCTs) that compared digital education interventions on any pediatric-related topic for postregistration health professions with traditional learning, no intervention, or other forms of digital education [37]. Eligible studies had to report at least one of the specified primary or secondary outcomes. Primary outcomes (measured using any validated or nonvalidated instruments) were (1) participants’ knowledge scores, (2) participants’ skills, and (3) participants’ attitudes toward the interventions or toward new clinical knowledge. Secondary outcomes included participants’ satisfaction with the intervention, participants’ change in clinical practice, the economic impact of digital education (eg, cost and cost effectiveness), patient-related outcomes, and any adverse or unintended effects of digital education.

### Search Strategy and Data Sources

We developed a comprehensive search strategy for 7 electronic databases: MEDLINE (Ovid), Embase (Elsevier), Cochrane Central Register of Controlled Trials (Wiley), PsycINFO (Ovid), Educational Research Information Center (Ovid), Cumulative Index to Nursing and Allied Health Literature (Ebsco), and Web of Science Core Collection (Thomson Reuters). The databases were searched for articles published from January 1990 to August 2017 without language or publication restrictions; studies published prior to this were not considered due to technological advances (Multimedia Appendix 1). We searched reference lists of all the studies that we deemed eligible for inclusion in our review and relevant systematic reviews. We also searched the International Clinical Trials Registry Platform Search Portal and metaRegister of Controlled Trials to identify unpublished trials.

### Data Collection and Analysis

The search results from different electronic databases were combined in a single Endnote library, and duplicate records were removed. Three review authors (SB, BMK, and LL) independently screened titles and abstracts of all the records to identify potentially eligible studies. We retrieved full-text copies of the articles deemed potentially relevant. Finally, three reviewers (SB, BMK, and LL) independently assessed the full-text versions of the retrieved articles against the eligibility criteria. Any disagreements were resolved through discussion between the reviewers.

### Data Extraction and Management

Each manuscript was independently extracted by two reviewers from a team of three (SB, BMK and LL). We extracted relevant characteristics related to types of participants (ie, doctors, nurses, or midwives), interventions used, comparators or control groups, outcome measures including details of assessment methods, and results from all included studies using a standard data collection form built on an Excel (Microsoft Corp) template (Multimedia Appendix 2). Any disagreements between the reviewers were resolved by discussion. We contacted study authors for any missing information, particularly information required to judge the risk of bias.

### Measures of Treatment Effect

For continuous outcomes, we extracted mean postintervention scores, standard deviations (SD), and number of participants for each intervention group and control groups. We used these data to calculate standardized mean differences (SMD) with associated 95% confidence intervals using random effect models. For studies that reported only median and ranges, we converted these to mean and SD [38]. Dichotomous data were extracted as number of events and number of participants in each intervention group. These data were used to calculated odds ratios (ORs) with associated 95% confidence intervals using random effect models. We were unable to identify a clinically meaningful interpretation of effect size in the literature for digital education interventions. Therefore, in line with other studies in the field, we presented outcomes using postintervention SMD and interpreted the effect size using the Cohen rule [39,40].

### Assessment of Risk of Bias in Included Studies

Three reviewers (SB, BMK, and LL) independently assessed the methodological risk of bias of included studies in line with the Cochrane methodology [40]. This includes domains covering random sequence generation, allocation concealment, blinding (outcome assessment), completeness of outcome data (attrition bias), selective outcome reporting (relevant outcomes reported) and other sources of bias such as baseline imbalances, inappropriate administration of an intervention, and contamination.

For cluster RCTs (cRCTs), we also assessed the risk of the following additional domains: recruitment bias, baseline imbalance, loss of clusters, incorrect analysis, and comparability with individual RCTs as recommended by Puffer et al [41]. Judgments concerning the risk of bias for each study were scored as high, low, or unclear. Disagreements were resolved by consensus between the two authors or through discussion with a third author (Multimedia Appendix 3) [41].

### Data Synthesis

We grouped studies by type of digital education, comparator group, and outcome. Where sufficient data were available, we used random effect meta-analysis to estimate summary effect estimates. Heterogeneity was assessed visually using forest plots and statistically using the *I*^2^ statistic [42]. Where meta-analysis was not possible (except for skill outcome), a narrative synthesis was presented. We aimed to carry out prespecified subgroup analysis including the analysis based on country income such as LMIC versus HIC (high-income countries). However, due to the limited primary data, we were unable to conduct prespecified subgroup analyses.

We also prepared summary of findings tables for the major comparisons focusing on high-fidelity mannequins and computer-based education and assessed the overall quality of evidence by using GRADEprofiler (GRADEproGDT Web-based version, McMaster University) [43]. We presented the findings of the review based on Preferred Reporting Items for Systematic Reviews and Meta-Analyses statements, and details of the statements are presented in Multimedia Appendix 4. Descriptions of the terminologies used in this review are presented in Multimedia Appendix 5.

## Results

### Search Results

**Figure 1:**
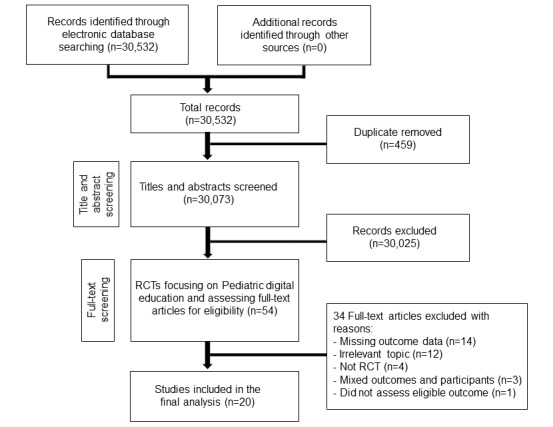
Flow of studies through the review process.

We used a common search strategy for a series of systematic reviews evaluating the effectiveness of digital education on different areas of health professions education. The overall searches identified 30,532 references. After reading titles and abstracts, 3466 references with different digital education interventions focusing on different areas of health professions education were identified, of which 54 studies focusing on pediatric education were selected for full-text review, and 20 trials fulfilled our inclusion criteria (1382 participants): 18 RCTs and two cRCTs (Figure 1).

Three studies were performed in the LMICs India [44], Ethiopia [45], and Lebanon [23]. The remaining were performed in HICs: North America (13 studies), Europe (3 studies), and Asia (1 study). Digital education was used for education in a variety of pediatric fields (Multimedia Appendix 6): neonatal/pediatric resuscitation and intubation (8 studies) [21-23,44-48], childhood obesity (2 studies) [49,50], pediatric emergency (1 study) [51], firearm injury prevention (1 study) [52], detection of childhood abuse (1 study) [53], drug prescriptions (1 study) [54], pediatric sedation (1 study) [55], child emergency nursing care (1 study) [56], counseling for parent’s smoking cessation (1 study) [57], pediatric orthopedic surgery (1 study) [58], and asthma (1 study) [59].

Participants in included studies were pediatricians (9 studies) [23,46,48,50-52,55,59,60]; postregistration nurses (4 studies) [22,44,53,56]; midwives and health extension workers (1 study) [45]; childcare health consultants (1 study) [49]; orthopedic surgery residents (1 study) [58]; family medicine residents (1 study) [47]; junior doctors (1 study) [54]; mixed participants including doctors, nurses, emergency technicians, and paramedics (1 study) [21]; and pediatric respiratory therapists and nurses (1 study) [57].

Seventeen studies were 2-arm studies: 9 compared digital education with traditional learning [21-23,44,45,47,48,56,58], 7 compared digital education with no intervention [50-54,57,59], and one compared 2 digital education methods [55]. Three studies included 3 intervention arms: 2 compared digital education with traditional learning and blended learning [46,60], and one compared digital education with traditional learning and no intervention [49]. Digital education technologies evaluated included high-fidelity mannequins (6 studies), computer-based education (12 studies), mLearning (1 study), and a virtual reality environment (1 study).

### Risk of Bias Assessment and Quality of Evidence

**Figure 2:**
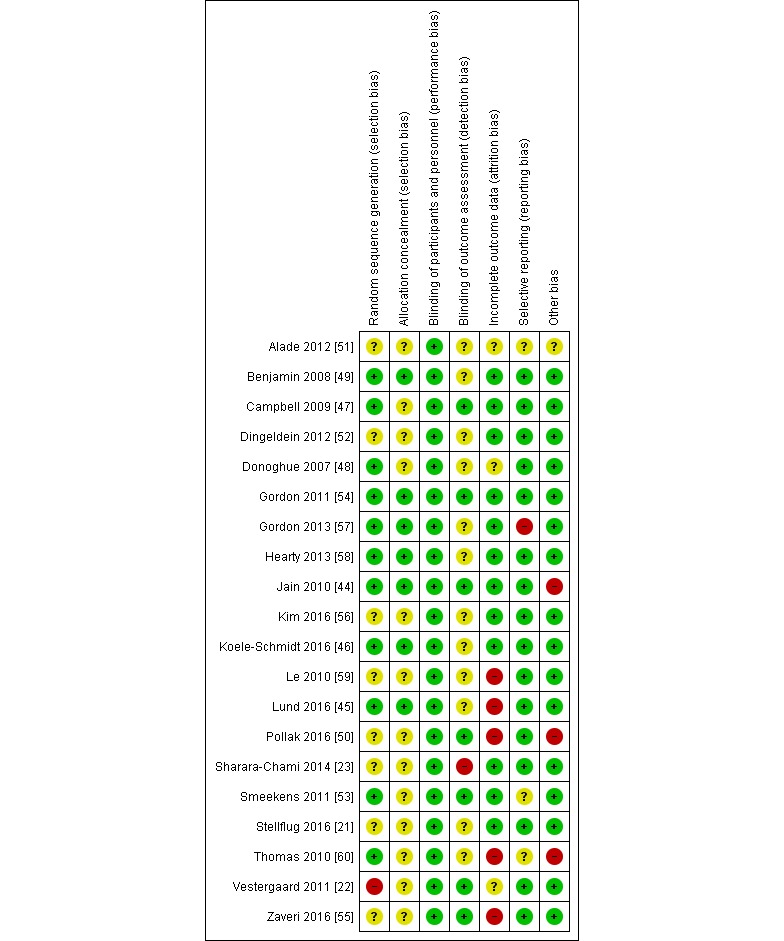
Risk of bias summary: review authors' judgements about each risk of bias item across all included studies.

The main limitation with the included studies was incomplete outcome data—5 studies were judged as high risk of bias for this domain [45,50,55,59,60], and for 3 studies, there was insufficient information on missing data to make a judgement [22,48,51]. Many studies were poorly reported, making it difficult to judge risk of bias. Randomization, concealment of allocation, and blinding of outcome assessors were poorly reported, with 8, 13, and 12 studies, respectively, judged at unclear risk of bias for these domains. However, only single studies were judged as high risk of bias for randomization [22], blinding of outcome assessment domains [23], selective reporting [57], and other bias [44]. Additional domains assessed for the 2-cluster trials were all at low risk of bias (Figure 2). The quality of evidence was low due to study inconsistencies, limitations, and/or imprecision across the studies (Multimedia Appendix 7).

**Figure 3:**
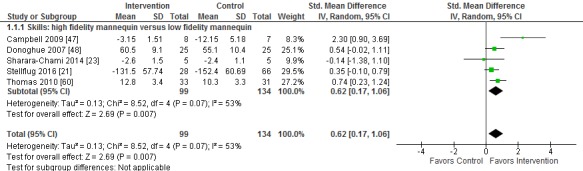
Difference in postintervention skill between interventions using high-fidelity mannequins and low-fidelity mannequins.

**Figure 4:**
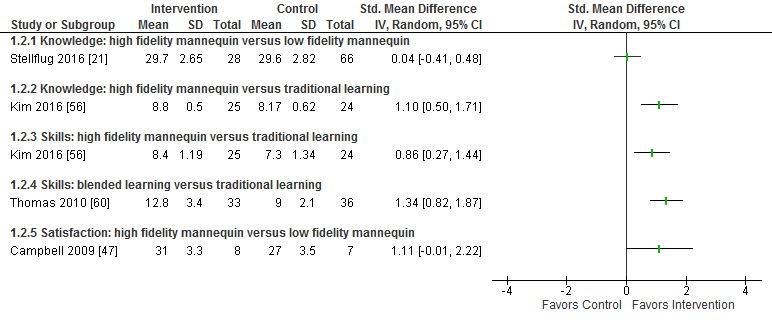
Difference in knowledge, skill, and satisfaction between interventions using high-fidelity mannequins and control groups.

**Figure 5:**
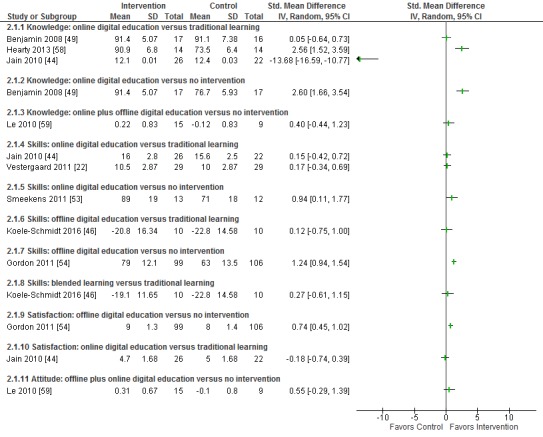
Difference in knowledge, skill, attitude, and satisfaction between interventions using computer-based education and control groups.

The detailed results of included studies are presented in Multimedia Appendix 8. Where available, standardized mean differences are presented in Figures 3-5.

### High-Fidelity Mannequins

Six studies (320 participants) assessed the use of high-fidelity mannequins to provide training in neonatal or pediatric resuscitation, 5 compared with low-fidelity mannequins [21,23,47,48,60] and one compared with traditional learning with checklist procedure training [56]. One study included an additional intervention group consisting of blended learning where participants receiving training used high-fidelity mannequins combined with team training [60].

All studies assessed participants’ psychomotor skill scores, although different measures of skill were used including time to complete different steps of the intubation and resuscitation, number of redirections provided during the procedure, and performance checklists for different tasks. Overall, high-fidelity mannequins were associated with greater postintervention skill scores compared with low-fidelity mannequins (SMD 0.62; 95% CI 0.17 to 1.06; *I*^2^=53%, 5 studies; Figure 3). One study compared high-fidelity mannequins with traditional learning with checklist procedure training and reported higher postintervention skill scores in the intervention group (SMD 0.86; 95% CI 0.27 to 1.44; Figure 4) [56]. The study that included an additional blended learning group reported greater improvement in skill in the blended learning group compared with low-fidelity mannequins alone (SMD 1.34; 95% CI 0.82 to 1.87) [60].

Two studies assessed knowledge [21,56]. One found that knowledge gain was greater with high-fidelity mannequins than with traditional learning (SMD 1.10; 95% CI 0.50 to 1.71). The other found no difference between groups (SMD 0.04; 95% CI –0.41 to 0.48). One study assessed participant satisfaction and found weak evidence for greater satisfaction with high-fidelity mannequins compared with low-fidelity mannequins (SMD 1.11; 95% CI –0.01 to 2.22).

### Computer-Based Education

Twelve studies (854 participants) assessed computer-based education, 7 compared with no intervention, 3 compared with traditional learning, one compared with traditional learning and no intervention, and one compared with blended learning and traditional learning. Nine studies evaluated computer-based online education including Web-based modules, tele-education, PowerPoint presentations, and online modules with audio-recorded patient cases. Two studies assessed offline computer-based education using PowerPoint presentations and computer-based video teaching. One assessed both online and offline digital education (Web- or CD-ROM–based learning plus 2 conference calls).

Seven studies (442 participants) assessed participant psychomotor skills postintervention [22,44,46,50,51,53,54]. The studies used different types of outcome measures such as time to complete different steps of the intubation and resuscitation, number of redirections provided during the procedure, and performance checklist for different tasks. Three studies compared computer-based education (online, tele-education, online video learning, and offline computer module with pictures and videos) with traditional learning [22,44,46]; one study included an additional blended learning group [46]. None of the comparisons suggested a difference in postintervention skill between learning groups (Figure 5). Two studies [53,54] compared computer-based learning (PowerPoint presentation, software, flash program, videos, animations, and online webpage) with no intervention, all reported greater improvement in skill following computer-based learning (Figure 5). Two studies did not report data in an appropriate form to include on the forest plot. One compared digital education (bimonthly, brief Web-based computer modules) with traditional learning to improve ultrasonography skills for pediatric emergencies and reported higher scores in the online learning group compared with traditional learning (*P*=.02) [51]. One study assessed primary care physicians’ adolescent weight management skills and favored digital education (online learning containing patients’ audio clips) over traditional learning (*P*=.001) [50].

Five studies (244 participants) assessed the effect of computer-based learning on knowledge [44,49,52,58,59]. One 3-arm study compared online education that included Web-based training on childhood weight management with traditional learning and no intervention. This study found no difference between online digital education and traditional learning (SMD 0.05; 95% CI –0.64 to 0.73) but reported greater knowledge scores following online digital education compared with no intervention (SMD 2.60; 95% CI 1.66 to 3.54) [49]. A further study that compared online and offline digital education with no intervention did not find any difference in knowledge between groups (SMD 0.40; 95% CI –0.44 to 1.23) [59]. Two studies compared online learning with traditional learning [44,58]. One found greater knowledge scores with online learning (SMD 2.56; 95% CI 1.52 to 3.59) [58] and the other found better outcome in the control group at postintervention with adjusted pretest values (SMD –13.68; 95% CI –16.59 to –10.77). However, it showed no difference between groups with unadjusted pretest values (SMD 0.17; 95% CI –0.40 to 0.74) [44]. One study that compared online learning with no intervention only reported knowledge outcomes for the intervention group [52].

Three studies (333 participants) provided information on attitude [52,57,59]. One study provided sufficient data to calculate SMDs; this study found no difference in attitude toward the intervention between digital education and no intervention groups (SMD 0.55; 95% CI –0.29 to 1.39) [59]. Two studies only reported information on attitude toward the intervention in the online learning group, with both suggesting positive attitudes toward online digital education [52,57].

Six studies (615 participants) provided information on satisfaction, although only 2 reported data in a suitable format to allow inclusion in the forest plot (Figure 5). One study reported greater satisfaction with offline digital education compared with no intervention (SMD 0.74; 95% CI 0.45 to 1.02) [54], and one study reported that participant satisfaction was similar for online digital education and traditional learning (SMD –0.18; 95% CI –0.74 to 0.39) [44]. Four studies only reported data for the intervention groups and reported that participants were satisfied with online and offline digital learning in terms of duration of the intervention, usability, format, and design [52,57-59].

### Mobile Learning

One study (176 participants) compared mLearning using a smartphone-based mobile app software with traditional learning to provide training in neonatal resuscitation to midwives and health extension workers. The study reported greater knowledge (SMD 0.91; 95% CI 0.60 to 1.22) and skill (SMD 0.99; 95% CI 0.68 to 1.30) scores in the mLearning group [45]. The study also assessed the impact on patient outcomes and found no difference in perinatal mortality rate between learning groups (OR 0.60; 95% CI 0.35 to 1.03).

### Virtual Reality Environments

One small study (32 participants) compared a virtual reality environment (ie, commercial virtual learning platform, SecondLife, which provides computer-generated virtual patient scenarios for clinical cases management) with online digital education to provide training in pediatric sedation to pediatric residents. This study found no difference in skill (SMD 0.24; 95% CI –0.47 to 0.94) [55] or knowledge (*P*=.14) between groups. None of the included studies reported adverse or unintended effects of digital education interventions or economic outcomes.

## Discussion

### Principal Findings

We identified 20 studies assessing the effects of digital interventions for education in the field of pediatrics for postregistration health professionals. Included studies assessed a broad range of interventions, comparisons, and outcomes. All studies reported that digital education was either as effective as or more effective than the control intervention for outcomes including skill, knowledge, attitude, and satisfaction. Only one study with high risk of other bias due to baseline imbalances suggested that outcomes were worse with digital education compared with standard learning. All studies reported data on either skill or knowledge. One study reported physicians’ change in practicing behavior and found similar effects between offline plus online digital education and no intervention. The only study to assess impact on patient outcome found no difference between intervention and control groups. The risk of bias was mainly unclear or high and the quality of evidence was low due to study inconsistencies, limitations, and/or imprecision across the included studies.

Studies assessed the use of different forms of digital education technologies such as high-fidelity mannequins (30%), computer-based education (60%), mLearning (5%), and virtual reality environment (5%). The majority of participants included in the studies were pediatric residents and junior doctors. Only four studies focused on nurses and one on midwives.

### Strengths and Limitations

Our systematic review has a number of strengths. We followed Cochrane guidance to minimize the risk of bias in the review process [40]. We used the Grading of Recommendations, Assessment, Development, and Evaluations (GRADE) criteria and assessed the quality of evidence for each primary and secondary outcome for the comparison with high-fidelity mannequin and low-fidelity mannequin. We conducted a comprehensive search across a broad range of databases to identify relevant studies. We searched for studies going back to 1990 as we considered that studies published before this were unlikely to be applicable to current practice due to technological advances. We included any postregistration health professionals working in the field of pediatrics to cover all available evidence on different types of participants on the topic. We also covered studies of any type of digital education interventions, which are primarily designed to deliver learning contents for pediatric education to synthesize the most robust evidence on the use of digital education for pediatric education. We believe that covering different types of health professions using different digital education technologies for pediatric education would provide the most comprehensive evidence on the topic. We conducted a formal risk of bias assessment to identify potential sources of bias in the primary studies. Two independent reviewers were involved in all stages of the review process to minimize the risk of bias and errors.

The small number of included studies meant that it was not possible to carry out any subgroup analyses or assess the risk of publication bias. Therefore, there is likelihood of publication bias, and the chances of publication bias cannot be ruled out in this case. The only comparison for which sufficient data were available to estimate summary effect sizes was for the impact of high-fidelity mannequins compared with low-fidelity mannequins on skill. Differences in interventions evaluated, populations targeted, and outcomes assessed also precluded meta-analysis for other outcomes and types of digital education.

The main methodological limitation of the included studies identified by our risk of bias assessment was the large number of withdrawals in 5 of the included studies. Almost half of the studies (9 out of 20) did not report on methods of randomization or allocation concealment and so it was not possible to judge whether appropriate steps were taken to minimize the risk of bias for these domains. Details of the intervention were often poorly reported, and most studies used a nonvalidated instrument to assess outcomes. Some studies did not assess skill level before the intervention so an imbalance across groups cannot be excluded. None of the studies reported following the Consolidated Standards on Reporting Trials (CONSORT) statement [61] or any other reporting guidelines. Twelve out of 20 studies included fewer than 50 participants, which meant they were unlikely to have sufficient power to identify differences between intervention groups. Only 3 of the 20 included studies were conducted in LMICs, thus the results of our review may have limited applicability for policymakers in in these countries. There was limited information on outcomes such as attitude, satisfaction, patient outcomes, costs, and adverse or untoward effects of digital education.

We are not aware of any other systematic reviews that focus on the effectiveness of digital education interventions for health professions in the field of pediatrics. Our review highlights the most up-to-date and comprehensive evidence regarding the effectiveness of digital education on the topic.

Further primary studies are needed to assess the impact of digital education for continuation of education for health professionals in the field of pediatrics. Studies should compare digital education with traditional face-to-face learning rather than with no intervention. Possibilities to continue education of health care workers in LMICs are limited, and it is known that the level of knowledge and skills is lower than the level of postgraduate health care workers in HICs [62,63]. Therefore, it could be argued that digital education interventions could be advantageous in these settings. However, only 3 out of the 20 studies were conducted in these settings. With limited studies from LMIC and poor quality of evidence for reported outcomes, this means that the applicability of the evidence from our review might be limited for policymakers implementing health policies in these countries. Moreover, there is limited information on other outcomes such as attitude, satisfaction, costs, and adverse or untoward effect of digital education interventions.

### Conclusion

Digital education for postregistration health professionals in pediatrics is at least as effective as traditional learning and more effective than no learning. High-fidelity mannequins were found to be more effective at improving psychomotor skills than traditional learning with low-fidelity mannequins. Computer-based offline/online digital education was better than no intervention for knowledge and skill outcomes and as good as traditional face-to-face learning. The evidence on other outcomes and other digital education modalities was limited. This review highlights evidence gaps in the field of digital education for health professions calling for more methodologically rigorous RCTs on the effectiveness of other forms of digital education such as mLearning, virtual reality environments, virtual patient scenarios, serious gaming and gamification, and massive open online courses for education of pediatric health professionals.

## Appendix

Multimedia Appendix 1 MEDLINE (Ovid) search strategy.

Multimedia Appendix 2 Data extraction form.

Multimedia Appendix 3 Risk of bias assessment for cluster randomized controlled trials.

Multimedia Appendix 4 Preferred Reporting Items for Systematic Reviews and Meta-Analyses checklist.

Multimedia Appendix 5 Glossary.

Multimedia Appendix 6 Characteristics of studies.

Multimedia Appendix 7 Summary of findings table: effects of high fidelity mannequin on knowledge, skills, attitude, satisfaction, and behavior change outcomes.

Multimedia Appendix 8 Results of the included studies.

## Abbreviations

CME: continuing medical education
CONSORT: Consolidated Standards of Reporting Trials
cRCT: cluster randomized controlled trial
GRADE: Grading of Recommendations, Assessment, Development, and Evaluations
HIC: high-income countries
LMIC: low- and middle-income countries
OR: odds ratio
RCT: randomized controlled trial
SMD: standardized mean difference
