# Supplementary material for: Digital Health Professions Education in the Field of Pediatrics: Systematic Review and Meta-Analysis by the Digital Health Education Collaboration
Source: J Med Internet Res. 2019 Sep 25;21(9):e14231. doi: 10.2196/14231 (PMC6785725; doi:10.2196/14231)
Supplement: Multimedia Appendix 2 [file jmir_v21i9e14231_app2.pdf]

	Study ID	
2. Study Identification	2.1. Journal where the study was published	
	2.2. Type of publication	
	2.3. Authors' affiliation	
3. Study design	3.1. Study design as specified in the report	
	3.2. Study aims & objectives	
	3.3. Countries where the study was conducted	
	3.4. WHO region	
	3.5.World Bank income category	
	3.6. Study start date	
	3.7. Study end date	
	3.8. Method of comparison	
4. Participants	4.1. Total number of participants invited to take part in the study	
	4.2. Total number of participants who agreed to take part in the study	
	4.3. Total number of participants meeting the inclusion criteria for participation in the study	
	4.4. Total number of participants included in the study	
	4.5. If cluster RCT, total number of clusters initially included in the study	
	4.6. If cluster RCT, total number of clusters randomised	
	4.7. Inclusion criteria	
	4.8. Exclusion criteria	
5. Intervention & Comparison	5.1. Total number of experimental groups (including the control group)	
	5.2. Were groups tested for baseline differences?	
	5.2.1. If there were baseline differences, please specify what the difference was	
	5.3. Indicate the type of degree or qualification that participants were pursuing	
	If other, please specify:	
	5.4. Year of study within the anticipated degree or qualification	
	**5.5. Control group**	

	5.5.1. Total number of participants/clusters allocated to the control group	
	5.5.2. Mean age (standard deviation) of the participants in the control group	
	5.5.3. Name of educational intervention used as control	
	5.5.4. Description of the control condition	
	5.5.5. Field of study	
	5.6.6. Exposure to the control condition during the whole study	
	5.5.7. Total exposure time to the intervention	
	5.5.8. Type of technology/devices used to deliver the intervention	
	5.5.9. Delivery approach of the intervention *If other, please specify:*	
	5.5.10. Was the usual delivery mode of the assessment changed?	
	5.5.11. If yes, please specify	
	5.5.12. Was the delivery mode of the assessment uniform across all the experimental groups?	
	**5.6. Intervention group I**	
	5.6.1. Total number of participants/clusters allocated to this intervention group.	
	5.6.2. Mean age (standard deviation) of the participants in this intervention group	
	5.6.3. Name of educational intervention used in this intervention group	
	5.6.4. Description of this intervention condition	
	5.6.5. Field of study	
	5.6.6. Exposure to this intervention condition during the whole study	
	5.6.7. Total exposure time to the intervention	
	5.6.8. Type of technology/devices used to deliver the intervention	
	5.6.9. Delivery approach of the intervention *If other, please specify:*	
	5.6.10. Was the usual delivery mode of the assessment changed?	

	5.6.11. If yes, please specify	
	5.6.12. Was the delivery mode of the assessment uniform across all the experimental groups?	
6. Outcomes	6.1. Was 'Knowledge' measured? - If not, please go to section 6.2.	
	6.1.1. Instrument or measure used to asses knowledge - as specified by the study authors	
	6.1.2. Is this a validated instrument?	
	6.2. Were 'Skills' measured? - If not, please go to section 6.3.	
	6.2.1. Instrument or measure used to asses skills - as specified by the study authors	
	6.2.2. Is this a validated instrument?	
	6.3. Were 'Attitudes' measured? - If not, please go to section 6.4.	
	6.3.1. Instrument or measure used to asses attitudes - as specified by the study authors	
	6.3.2. Is this a validated instrument?	
	6.4. Was 'Student satisfaction' measured? - If not, please go to section 6.5.	
	6.4.1. Instrument or measure used to asses student satisfaction - as specified by the study authors	
	6.4.2. Is this a validated instrument?	
	6.5. Was an economic evaluation of the eLearning intervention performed?	
	6.5.1. Were quantitative indicators like costs, investments, hardware, software, license fees and benefits/savings of the eLearning intervention measured?	
	6.5.2. Was the urgency of the eLearning intervention (i.e., due to a new regulation or organisational demand) mentioned?	
	6.5.3. Were qualitative-strategic indicators of the eLearning intervention like quality and performance improvements measured?	

	6.5.4. Were external factors of the eLearning intervention like synergy effects or economies of scope measured?	
	6.5.5. Please list any additional economic indicators that were measured	
9. Study findings	**9.1. Control group**	
	9.1.1. Outcome reported	
	9.1.2. Measure of effect size (as measured by the study authors)	
	9.1.3. Measure of dispersion (as measured by the study authors)	
	9.1.1. Outcome reported	
	9.1.2. Measure of effect size (as measured by the study authors)	
	9.1.3. Measure of dispersion (as measured by the study authors)	
	9.1.1. Outcome reported	
	9.1.2. Measure of effect size (as measured by the study authors)	
	9.1.3. Measure of dispersion (as measured by the study authors)	
	9.1.1. Outcome reported	
	9.1.2. Measure of effect size (as measured by the study authors)	
	9.1.3. Measure of dispersion (as measured by the study authors)	
	If more than one outcome was reported, please insert more cells here and copy and paste the relevant data entry boxes.	
	**9.2. Intervention I group**	
	9.2.1. Outcome reported	
	9.2.2. Measure of effect size (as measured by the study authors)	
	9.2.3. Measure of dispersion (as measured by the study authors)	
	9.2.1. Outcome reported	
	9.2.2. Measure of effect size (as measured by the study authors)	
	9.2.3. Measure of dispersion (as measured by the study authors)	

	9.2.1. Outcome reported	
	9.2.2. Measure of effect size (as measured by the study authors)	
	9.2.3. Measure of dispersion (as measured by the study authors)	
	9.2.1. Outcome reported	
	9.2.2. Measure of effect size (as measured by the study authors)	
	9.2.3. Measure of dispersion (as measured by the study authors)	
	If more than one outcome was reported, please insert more cells here and copy and paste the relevant data entry boxes.	
	**9.5. Comparison I**	
	9.5.1. Please indicate the intervention groups being compared	
	9.5.2. Please indicate the outcomes being compared	
	9.5.3. Statistical test used for the comparison	
	9.5.4. Result of the test	
	9.5.5. P value / Confidence intervals	

10. ImplementatioNAdoption of eLearning interventions	
	10.1. Organizational setting
	10.2. Technological infrastructure
	10.3. Instructional Systems Design and Curriculum development
	10.4. Delivery

	10.5. Advantages of eLearning - as reported by the study authors	
	10.6. Disadvantages of eLearning - as reported by the study authors	
**11. Sustainability of eLearning interventions**	11.1. Source of financing - as reported by the study authors	
	11.2. Did the intervention undergo a formal accreditation process within the host institution?	
	11.3. If yes, please describe	

	11.4. Was the eLearning intervention developed for this study consequently adopted as a formal method for the delivery of education at the host institution?	
	11.5. If yes, please specify	
12. Miscellanous	12.1. Study conclusions - as stated by the study authors	
	12.2. Limitations of the study - as reported by the study authors	
	12.3. Was contact with the study authors sought? - If No, please go to section 12.5	
	12.4. Please indicate the nature of the information requested from the study authors	
	12.5. Please indicate the results of the request for information	

	12.6. Additional notes	
