# Supplementary material for: Digital Health Professions Education in the Field of Pediatrics: Systematic Review and Meta-Analysis by the Digital Health Education Collaboration
Source: J Med Internet Res. 2019 Sep 25;21(9):e14231. doi: 10.2196/14231 (PMC6785725; doi:10.2196/14231)
Supplement: Multimedia Appendix 3 [file jmir_v21i9e14231_app3.pdf]

Multimedia Appendix 3: Risk of bias assessment for cluster RCT

**Study ID**	**Recruitment bias**	**Baseline imbalance**	**Loss of clusters**	**Incorrect analysis**	**Comparability with individual trials**
Lund 2016	Low	Low	Low	Low	Unclear
	Recruitment of participants were carried out before randomisation of clusters.	No baseline difference was reported	3 clusters were excluded before randomisation and loss of cluster was not reported after randomisation.	The study carried out the analyses based on the intention-to-treat principle, and all available data were included in the analysis.	Comparison between cRCT and RCT could not be made due to heterogeneity in the specialization of participants and interventions.
Stellflug 2016	Low	Low	Low	Low	Unclear
	Participants signed up for paediatric advanced life supported were asked to participate in the study. Recruitment was carried out before randomisation.	No baseline difference was reported	Loss of clusters were not reported.	The study used proper methods to handle the data and carried out the analysis.	Comparison between cRCT and RCT could not be made due to heterogeneity in the specialization of participants and interventions.
