# Supplementary material for: Digital Health Professions Education in the Field of Pediatrics: Systematic Review and Meta-Analysis by the Digital Health Education Collaboration
Source: J Med Internet Res. 2019 Sep 25;21(9):e14231. doi: 10.2196/14231 (PMC6785725; doi:10.2196/14231)
Supplement: Multimedia Appendix 5 [file jmir_v21i9e14231_app5.pdf]

Multimedia Appendix 5: Glossary

Glossary	Definitions/Descriptions
Fidelity, High-Fidelity Mannequin and Low-Fidelity Mannequin	"Fidelity can be defined as the extent to which the skills of the real task are captures by the simulated task. High fidelity or state of the art fidelity mannequins usually include a complex scenario in which computer-aided mannequins interact with the trainees. For low fidelity mannequins, it is meant for demonstrating a simple skills such as performing cardio-pulmonary resuscitation or intubation for air-way management [1]."
Offline (and computer-based) digital education	Offline digital education can be defined as offline and stand-alone computer-based or computer-assisted learning where internet or intranet connection is not required for the learning activities [2].
Online (and computer-based) digital education	Online digital education or online learning can be defined as the interventions that require the use of a 'Transmission Control Protocol' (TCP) and an 'Internet Protocol' (IP) as a standard for the learning activities [3].
Virtual Reality (VR) Environment	VR is a technology that allows the user to explore and manipulate computer-generated real or artificial three-dimensional (3D) multimedia sensory environments in real time. It allows for a first-person active learning experience through different levels of immersion; that is, a perception of the digital world as real and the ability to interact with objects and/or perform a series of actions in this digital world [4].

References

1. Munshi F, Lababidi H, Alyousef S. Low- versus high-fidelity simulations in teaching and assessing clinical skills. J Taibah Univ Med Sci. 2015;10(1):12-5. doi: 10.1016/j.jtumed.2015.01.008
2. Kyaw BM, Posadzki P, Dunleavy G, Semwal M, Divakar U, Hervatis V. Offline digital education for medical students: systematic review and meta-analysis by the digital health education collaboration. J Med Internet Res 2019 Mar 25;21(3):e13165. PMID: 30907731 doi: 10.2196/13165
3. George PP, Zhabenko O, Kyaw BM, Antoniou P, Posadzki P, Saxena N. Online digital education for postregistration training of medical doctors: systematic review by the digital health education collaboration. J Med Internet Res 2019 Feb 25;21(2):e13269. PMID: 30801252 doi: 10.2196/13269
4. Kyaw BM, Saxena N, Posadzki P, Vseteckova J, Nikolaou CK, Pradeep P. Virtual reality for health professions education: a systematic review and meta-analysis by the digital health education collaboration. J Med Internet Res 2018. PMID: 30668519
