# Supplementary material for: Digital Health Professions Education in the Field of Pediatrics: Systematic Review and Meta-Analysis by the Digital Health Education Collaboration
Source: J Med Internet Res. 2019 Sep 25;21(9):e14231. doi: 10.2196/14231 (PMC6785725; doi:10.2196/14231)
Supplement: Multimedia Appendix 6 [file jmir_v21i9e14231_app6.pdf]

Multimedia Appendix 6. Characteristics of the studies

Study ID, Design, Country	Number of participants	Intervention	Control	Participants	Field of the study	Learning objectives
**Computer-Based Digital Education**						
Alade 2012 [51] RCT, USA	16	Online: bi-monthly brief web-based computer module on basic pediatric emergency ultrasonography	No intervention	Pediatrics and emergency medicine fellows; pediatricians practicing in emergency medicine	Pediatric emergency	To improve residents' knowledge on pediatric emergency ultrasonography and to promote knowledge retention on pediatric emergency ultrasonography
Benjamin 2008 [49] RCT, USA	51	Online: web-based training modules on basic nutrition and physical activity principles important for the promotion of healthy child weight	No intervention	Child Care Health Consultants	Childhood obesity	To improve participants' knowledge on overweight prevention including basic nutrition and physical activity principles important for the promotion of child healthy weight
Dingeldein 2012,[52] RCT, USA	92	Online: web-based curriculum on firearm injury prevention	No intervention	Pediatric residents	Firearm injury prevention	To improve pediatric residents' knowledge and skills on firearm injury prevention
Gordon 2013 [57] RCT, USA	217	Online: a 3-hour web-based tobacco cessation education program	No intervention	Pediatric respiratory therapists, nurses and	Counselling for parents smoking cessation	To improve participants' attitude and satisfaction with web-based tobacco

				nurse practitioners		cessation education program
Pollak 2016 [50] RCT, USA	46	Online: 60 minutes individually tailored, online module including audio-recorded patient cases	No intervention	Medical doctors (83% pediatricians)	Childhood obesity	To improve physicians' clinical management of overweight and obese adolescents
Smeekens 2011 [53] RCT, Netherlands	38	Online: web-based program on child abuse consisting of three different modules: recognition, acting and communication.	No intervention	Emergency department nurses	Childhood abuse	To improve detection of childhood abuse by ED nurses
Hearty 2013 [58] RCT, USA	28	Online: web-based module containing videos of surgical cases demonstrating different steps of surgery and components	Textbook-based study	Orthopedics surgery residents	Pediatric orthopedic surgery	To improve orthopedics residents' preparedness for closed reduction and pinning of pediatric supracondylar fractures
Jain 2010 [44] RCT, India	49	Online: web-based tele-education on neonatal resuscitation at the tertiary care center	Classroom teaching (face-to-face)	Nurses	Neonatal resuscitation	To improve nurses knowledge and skills of neonatal resuscitation

Benjamin 2008 [49] RCT, USA	51	Online: web-based training modules on the basic nutrition and physical principles important for the promotion of child healthy weight	classroom teaching (face-to-face)	Child Care Health Consultants who provide consultation to child care centers	Counselling on childhood obesity	To improve participants' knowledge on management of overweight children
Vestergaard 2011 [22] RCT, Denmark	58	Online: web-based training video on pediatric basic life support	Instructor-led mannequin training	Nurses (from pediatric and maternity wards)	Pediatric Basic Life Support	To improve nurses' pediatric basic life support or resuscitation skills with online education training
Gordon 2011 [54] RCT, USA	205	Offline: Computer training program including PowerPoint presentation, flash program, video and animations	No intervention	Junior doctors	Drug prescriptions	To improve junior doctors' prescribing skills and satisfaction
Koele-Schmidt 2016 [46] RCT, USA	30	Offline: standard computer modules involving a slide show of neonatal airway anatomy and technique for direct laryngoscopy	Standard bedside teaching	Pediatric and Anesthesia residents	Neonatal endotracheal intubation	To improve residents' neonatal endotracheal intubation skill with the use of offline digital education

Le 2010 [59] RCT, USA	24	Offline and Online: Web- or CD-ROM-based multimedia learning modules and two conference calls	No intervention	Pediatricians	Asthma learning program for pediatricians	To improve pediatricians' satisfaction and change in knowledge, attitude and treatment behavior regarding childhood asthma management
Koele-Schmidt 2016 [46] RCT, USA	30	Blended learning (standard teaching plus offline digital education)	Standard bedside teaching	Pediatric and Anesthesia residents	Neonatal endotracheal intubation	To improve residents' neonatal endotracheal intubation skill with offline digital education
**High Fidelity Mannequins (HFM)**						
Thomas 2010 [60] RCT, USA	100	High Fidelity Mannequin	1) Low fidelity mannequin or 2) Blended learning (Low fidelity mannequin plus team training)	Interns for pediatrics, internal medicine, family medicine, emergency medicine and gynecology	Neonatal resuscitation	To improve participants' teamwork and communication skills in neonatal resuscitation
Campbell 2009 [47] RCT, Canada	15	High Fidelity Mannequin	Low fidelity mannequin	Family medicine residents	Neonatal resuscitation	To improve residents' neonatal resuscitation skills with use of high fidelity mannequin
Donoghue 2009 [48] RCT, USA	50	High Fidelity Mannequin	Low fidelity mannequin	Pediatric residents	Pediatric Advanced Life Support	To improve residents' pediatric resuscitation

						skills with the use of high fidelity mannequin
Stellflug 2016 [21] cRCT, USA	94	High Fidelity Mannequin	Low fidelity mannequin	Medical doctors, nurses, emergency technicians and paramedics	Pediatric Advanced Life Support	To improve participants' knowledge retention and skill in pediatric advanced life support training
Sharara-Chami 2014 [23] RCT, Lebanon	11	High Fidelity Mannequin	Low fidelity mannequin	Pediatric residents	Pediatric intubation	To improve residents' skills in airway management and endotracheal intubation with the use of high fidelity mannequin
Kim 2016 [56] RCT, South Korea	50	High Fidelity Mannequin	Checklist procedure training	Nurses working in medicine, surgery wards or outpatient-clinic	Maternal and child emergency nursing care	To improve nurses' knowledge, attitudes and skills on maternal and child emergency care with the use of high fidelity mannequin
**Mobile learning (mLearning)**						
Lund 2016 [45] cRCT, Ethiopia	176	Mobile application or apps	Standard Care without the assistance of mobile apps	Midwife and health extension workers	Neonatal resuscitation	To improve health care workers' knowledge and skill in neonatal resuscitation and to improve perinatal survival with the use of the safe delivery app
**Virtual Reality Environment**						

Zaveri 2016 [55] RCT, USA	32	Virtual Reality Environment in SecondLife® which provides computer generated three-dimensional images of real life scenarios with or without immersion	Computer-based online digital education	Pediatric residents	Pediatric sedation	To improve pediatric residents' pediatric sedation knowledge and skills with the use of virtual reality environment
