# Supplementary material for: Digital Health Professions Education in the Field of Pediatrics: Systematic Review and Meta-Analysis by the Digital Health Education Collaboration
Source: J Med Internet Res. 2019 Sep 25;21(9):e14231. doi: 10.2196/14231 (PMC6785725; doi:10.2196/14231)
Supplement: Multimedia Appendix 7 [file jmir_v21i9e14231_app7.pdf]

Multimedia Appendix 7. Summary of Findings Table

**High fidelity mannequin compared with low fidelity mannequin or traditional learning**				
**Population**: doctors, family medicine residents, pediatricians, nurses, emergency technicians and paramedics<br><br>**Settings**: Hospitals or clinics<br><br>**Intervention**: high fidelity mannequins<br><br>**Comparison**: low fidelity mannequins, traditional learning with checklist procedure training				
**Outcomes**	**Illustrative comparative risks (95% CI)**	**Number of participants (number of studies)**	**Quality of the evidence (GRADE)**	**Comments**
**Skills** (measured with checklists), post-intervention	The mean skills score in high fidelity mannequin groups was 0.62 standard deviations higher (0.17 higher to 1.06 higher) compared to low fidelity mannequin.	320 students<br><br>(6 studies)	⊕⊕⊖⊖<br><br>Low [a,b]	The standard deviations was derived from the pooled estimate of five studies which compared high fidelity mannequin to low fidelity mannequin, a SMD of 0.62 (95% CI: 0.17 to 1.06) which indicates a moderate effect size [21, 23, 47, 48, 60]. The result of one study (50 participants) was not added to the meta-analysis as the study compared high fidelity mannequin to traditional learning with checklist procedural training. However, the study authors reported improved post-

				intervention skill scores in the intervention group compared to the control group [56].
**Knowledge** (measured with questionnaires and MCQs), post-intervention	Not estimable (see comment)	144 participants (2 studies)	⊕⊕⊝⊝ Low [a,b]	One study found that knowledge gain was greater with high fidelity mannequins than with traditional learning [56]. The other found no difference between groups [21].
**Satisfaction** (measured with 5-point Likert scale), post-intervention	Not estimable (see comment)	15 participants (1 study)	⊕⊕⊝⊝ Low [b,c]	One study assessed participants' satisfaction and found weak evidence for greater satisfaction with high fidelity mannequins compared to low fidelity mannequins [47].
**Attitude**	No studies reported attitude outcome.			
**Behavior change**	No studies reported behavior change outcome.			
**Adverse outcome**	No studies reported adverse events.			
**Economic evaluation**	No studies reported economic evaluation.			
OSCE-objective structured clinical examination; VP- virtual patient.				

GRADE Working Group grades of evidence
High quality: further research is very unlikely to change our confidence in the estimate of effect.
Moderate quality: further research is likely to have an important impact on our confidence in the estimate of effect and may change the estimate.
Low quality: further research is very likely to have an important impact on our confidence in the estimate of effect and is likely to change the estimate.
Very low quality: we are very uncertain about the estimate.

Legend:

[a] Downgraded by one level for study limitations: the risk of bias was unclear or high in most included studies.

[b] Downgraded by one level for inconsistency: the heterogeneity between studies was high with a lack of overlap among confidence intervals.

[c] Downgraded by one level for imprecision: number of participants (effective sample size) in the study is less than the number of patients generated by a conventional sample size calculation for a single adequately powered trial (optimal information size)
