# Supplementary material for: Digital Health Professions Education in the Field of Pediatrics: Systematic Review and Meta-Analysis by the Digital Health Education Collaboration
Source: J Med Internet Res. 2019 Sep 25;21(9):e14231. doi: 10.2196/14231 (PMC6785725; doi:10.2196/14231)
Supplement: Multimedia Appendix 8 [file jmir_v21i9e14231_app8.pdf]

Multimedia Appendix 8: Results of the included studies

Study ID	Intervention	Control	Knowledge	Skill	Attitude	Satisfaction	Patient-related outcome	Behaviour change practice
**Computer based online digital education (Online digital education)**								
Alade 2012	Online digital education	No intervention	-	Intervention MCS: 24.3 Control MCS: 12.5, P=0.02	-	-	-	-
Dingeldein 2012	Online digital education	No intervention	Intervention M (SD): 1.9 (0.74)	-	Intervention M: 2.7 (0.84)	Intervention: appropriate amount time for intervention (90% of participants); provides valuable information (98% of the participants); recommend to others (88% of the participants)	-	-
Gordon 2013	Online digital education	No intervention	-	-	Intervention CS: 4.07 Control CS: 3.82	Intervention M: 4	-	-
Pollak 2016	Online digital education	No intervention	-	MCS: 0.6; 95%CI (0.2,1.0); P = 0.001)	-	-	-	
Smeekens 2011	Online digital education	No intervention	-	Intervention M (SD): 89 (19) Control M (SD): 71 (18)	-	-	-	-
Benjamin 2008	Online digital education	No intervention	Intervention M (SD): 91.4 (5.07) Control M (SD): 76.7 (5.93)	-	-	-	-	-

	Online digital education	Traditional Learning	Intervention M (SD): 91.4 (5.07) Control M (SD): 91.1 (7.38)	-	-	-	-	-	-
Hearty 2013	Online digital education	Traditional Learning	Intervention M (SD): 90.9 (6.8) Control M (SD): 73.5 (6.4)	-	-	No numerical data available. The study provided the findings on different subtopics narratively	-	-	
Jain 2010	Online digital education	Traditional Learning	Intervention M (SD): 12.1 (0.01) Control M (SD): 12.4 (0.03)	Intervention M (SD): 16 (2.8) Control M (SD): 15.6 (2.5)	-	Intervention M (SD): 4.7 (0.25) Control M: 5	-	-	
Vestergaard 2017	Online digital education	Traditional Learning	-	Intervention M: 10.5 Control M: 10 P value: 0.51	-	-	-	-	
**High Fidelity Mannequin (HFM)**									
Campbell 2009	HFM	LFM	-	Intervention M (SD): -3.15 (1.51) Control M (SD): -12.15 (5.18)	-	Intervention M (SD): 31 (3.3) Control M (SD): 27 (3.5)	-	-	
Donoghue 2009	HFM	LFM	-	Intervention M (SD): 60.5 (9.1) Control M (SD): 55.1 (10.4)	-	-	-	-	
Kim 2016	HFM	Traditional Learning	Intervention M (SD): 8.8 (0.5) Control M (SD): 8.1 (0.62)	Intervention M (SD): 8.4 (1.19) Control M (SD): 7.3 (1.34)	-	-	-	-	

Sharara-Chami 2014	HFM	LFM	-	Intervention M (SD): -2.6 (1.5) Control M (SD): -2.4 (1.1)	-	-	-	-
Stellflug 2016	HFM	LFM	Intervention M (SD): 29.7 (2.65) Control M (SD): 29.6 (2.82)	Intervention M (SD): -131.5 (57.74) Control M (SD): -152.4 (60.69)	-	-	-	-
Thomas 2010	HFM	LFM	-	Intervention M (SD): 12.8 (3.4) Control M (SD): 10.3 (3.3)	-	-	-	-
	Blended Learning (HFM plus team training)	LFM	-	Intervention M (SD): 12.8 (3.4) Control M (SD): 9 (2.1)	-	-	-	-
**Computer based offline digital education (Offline digital education)**								
Gordon 2011	Offline digital education	No intervention	-	Intervention M (SD): 79 (12.1) Control M (SD): 63 (13.5)	-	Intervention M (SD): 9 (1.3) Control M (SD): 8 (1.4)	-	-
Koele-Schmidt 2016	Offline digital education	Traditional Learning	-	Intervention M (SD): -20.8 (16.34) Control M (SD): -22.8 (14.58)	-	-	-	-
	Blended Learning (Offline digital education plus traditional learning)	Traditional Learning	-	Intervention M (SD): -19.1 (11.65) Control M (SD): -22.8 (14.58)	-	-	-	-
**Computer-based offline plus online digital education (Offline and Online digital education)**								

Le 2010	Offline and Online digital education	No intervention	Intervention CS (SD): 0.22 (0.83) Control CS (SD): -0.12 (0.83)	-	Intervention CS (SD): 0.31 (0.67) Control CS (SD): -0.1 (0.8)	Intervention M (SD): 4.4 (3.49)	-	Intervention CS (SD): 8 (15.37) Control CS (SD): 8.69 (13.28)
**Mobile Learning (mLearning)**								
Lund 2016	mLearning	No intervention	Intervention M (SD): 5.9 (2.02) Control M (SD): 4.3 (1.45)	Intervention M (SD): 12.9 (5.92) Control M (SD): 7.5 (4.91)	-	-	Intervention (Events/Total): 21/1478 Control (Events/Total): 39/1665	-
**Virtual Reality Environment (VRE)**								
Zaveri 2016	VRE	Online digital education	Intervention Median (IQR): 1 (-1 to 2.25) Control Median (IQR): 3 (1 to 4), P=0.14	Intervention M (SD): 16 (2.05) Control M (SD): 15.2 (4.02)	-	-	-	-

HFM = High Fidelity Mannequin, LFM = Low Fidelity Mannequin, M = Post-test mean score, MCS = Mean change score between the groups, CS = Mean change score within the group, SD = Standard Deviation
